# Supplementary figures and images for: Small RNA, Transcriptome and Degradome Analysis of the Transgenerational Heat Stress Response Network in Durum Wheat
Source: Int J Mol Sci. 2021 May 24;22(11):5532. doi: 10.3390/ijms22115532 (PMC8197280; doi:10.3390/ijms22115532)

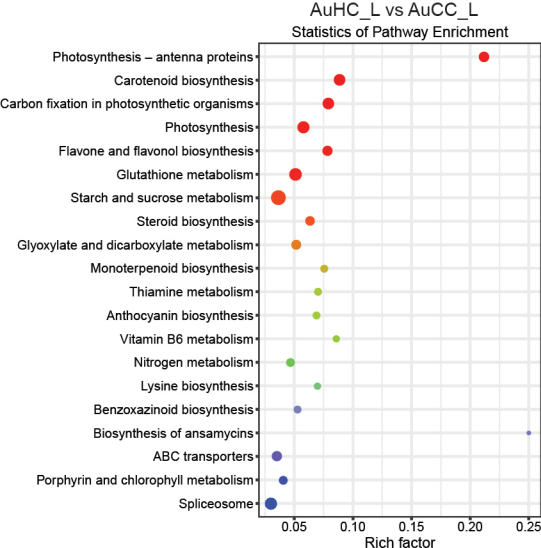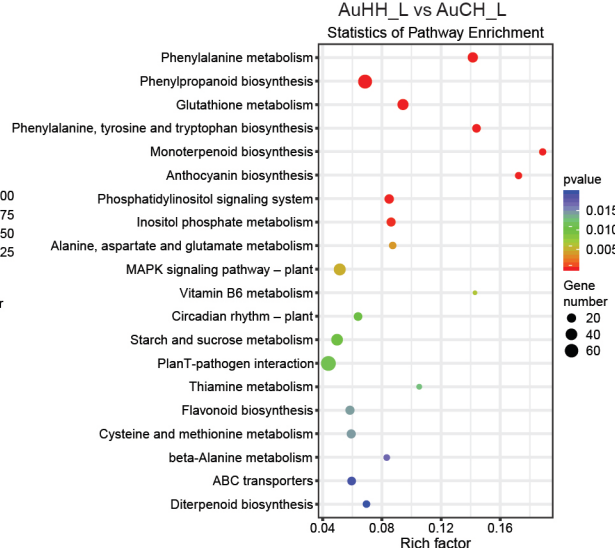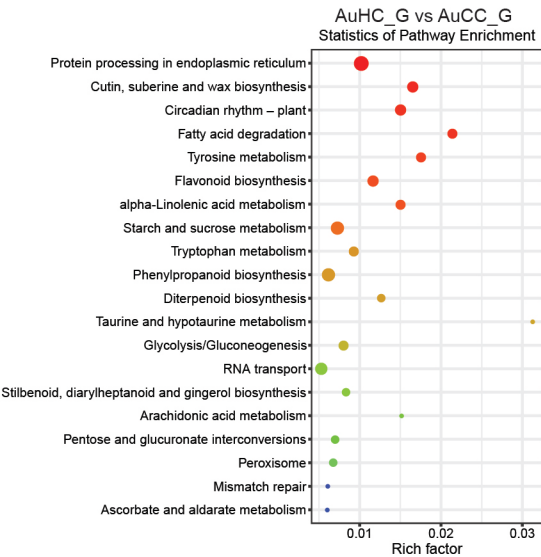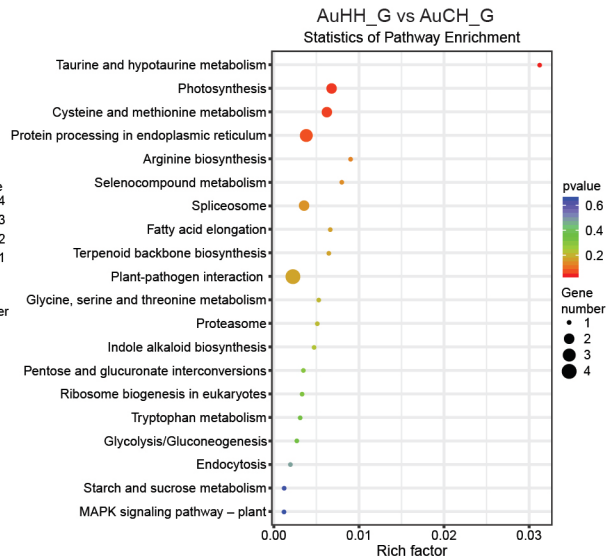

Supplement: Supplementary file 1 [file ijms-22-05532-s001.zip › Figure S1.pdf]
